# Endothelium-Dependent Nitric Oxide-Mediated Vasorelaxant Effects of BPC 157 in Human Internal Mammary Artery

**DOI:** 10.3390/jcm15093488

**Published:** 2026-05-02

**Authors:** Alperen Kutay Yildirim, Ahmet Onur Dastan, Meric Demeli Ertus, Mesher Ensarioglu, Kubilay Karabacak, Bilge Pehlivanoglu

**Affiliations:** 1 Department of Cardiovascular Surgery, Gulhane Training and Research Hospital, Ankara 06010, Turkey; 2 Department of Physiology, Faculty of Medicine, Hacettepe University, Ankara 06100, Turkey; ahmetdastan@hacettepe.edu.tr (A.O.D.); pbilge@hacettepe.edu.tr (B.P.); 3 Department of Physiology, Faculty of Medicine, Zonguldak Bulent Ecevit University, Zonguldak 67600, Turkey; mericdml@gmail.com; 4 Department of Anesthesiology and Reanimation, Gulhane Training and Research Hospital, Ankara 06010, Turkey; meshercapras@gmail.com; 5 Department of Cardiovascular Surgery, Gulhane Training and Research Hospital, University of Health Sciences, Ankara 06010, Turkey; kubilaykarabacak@yahoo.com

**Keywords:** BPC 157, internal mammary artery, L-NAME, vasodilation, nitric oxide

## Abstract

**Background/Objectives:** Body Protection Compound-157 (BPC 157) is a stable gastric pentadecapeptide with cytoprotective, pro-angiogenic, and nitric oxide (NO)-modulating properties that has gained increasing attention for its therapeutic potential. Although vasodilatory effects have been demonstrated in animal models, functional evidence in human arterial tissue remains limited. This study investigated the effects of BPC 157 on vascular tone in human internal mammary artery (IMA) rings and evaluated the contribution of endothelial NO signaling. **Methods:** Residual IMA segments obtained from elective coronary artery bypass graft surgeries (*n* = 12) were dissected into endothelium-intact and endothelium-denuded rings. Following equilibration, the rings were challenged by phenylephrine (PheE; 3 × 10^−6^ M) to induce contraction. Cumulative concentration–response curves of BPC 157 (0.01–1 mg/mL) for five consecutive doses were constructed. The involvement of NO was assessed by BPC 157 dose–response curves in the nitric oxide synthase (NOS) inhibitor Nω-nitro-L-arginine methyl ester (L-NAME; 10^−6^ M) pre-incubated rings. Maximum force of contraction, area under the curve, maximum response (Emax), and negative logarithm of the half-maximal effective concentration (pEC50) values were analyzed. **Results:** BPC 157 produced a concentration-dependent reduction in PheE-induced contraction in both groups, with significantly greater relaxation in endothelium-intact rings (*p* < 0.05). L-NAME increased contractile responsiveness in intact rings and attenuated BPC 157-induced relaxation. Under NOS inhibition, differences between groups progressively diminished and concentration–response curves converged at higher concentrations. Emax analysis demonstrated that endothelial integrity markedly enhanced maximal vasorelaxation, whereas this advantage was largely abolished after NOS inhibition. **Conclusions:** BPC 157 induces concentration-dependent vasorelaxation in human arterial tissue, predominantly mediated via an endothelium-dependent NO pathway. Endothelial integrity primarily enhances maximal efficacy, while residual effects indicate additional mechanisms. These findings provide early mechanistic evidence for the vascular activity of BPC 157, although further molecular and in vivo studies are required to clarify its clinical relevance.

## 1. Introduction

The internal mammary artery (IMA) is the preferred conduit for coronary artery bypass grafting (CABG) due to its superior long-term patency and resistance to atherosclerosis [1,2]. This advantage is largely attributable to the unique endothelial properties of the IMA, which is rich in endothelial nitric oxide synthase (eNOS) and releases substantially greater amounts of nitric oxide (NO) than other conduit vessels such as saphenous veins and carotid arteries [3,4,5,6,7]. Endothelium-derived NO is a principal mediator of vasodilation in the IMA, acting through the cyclic guanosine monophosphate (cGMP)–protein kinase G pathway to reduce intracellular calcium levels and induce vascular smooth muscle cell (VSMC) relaxation [7,8,9]. Additional endothelium-derived mediators also contribute to the regulation of IMA vascular tone. Prostacyclin (PGI_2_), produced via the cyclooxygenase pathway, induces relaxation through cyclic adenosine monophosphate (cAMP)–protein kinase A signaling [10,11,12]. Endothelium-derived hyperpolarizing factor (EDHF), identified in human IMA as 11,12-epoxyeicosatrienoic acid, promotes relaxation by activating large-conductance calcium-activated potassium (BKCa) channels on VSMCs [13,14,15]. Notably, vascular endothelial growth factor (VEGF)-induced relaxation in human IMA has also been shown to involve both NO and PGI_2_ pathways mediated through the kinase insert domain receptor (KDR)/vascular endothelial growth factor receptor 2 (VEGFR2) [16,17].

In addition to these endothelium-dependent mechanisms, non-endothelial pathways operating at the level of vascular smooth muscle also regulate IMA contractile tone. Contraction in the IMA is governed by the interplay between calcium-dependent myosin light chain kinase (MLCK) activation and Rho-associated kinase (ROCK)-mediated calcium sensitization of the contractile apparatus [2,18]. On the relaxation side, endothelium-independent vasorelaxation in the IMA can be mediated by activation of potassium channels on VSMCs—including voltage-gated potassium (Kv) channels, BKCa channels, and adenosine triphosphate (ATP)-sensitive K^+^ channels—which hyperpolarize the smooth muscle membrane and reduce calcium entry through voltage-operated calcium channels [19,20]. Both endothelium-dependent and endothelium-independent mechanisms are clinically relevant, as they represent potential therapeutic targets for the regulation of vascular tone.

Despite these multiple vasodilatory pathways, perioperative arterial spasm remains a clinically significant complication that can compromise graft flow and surgical outcomes [1,2]. The mechanism of IMA graft spasm is multifactorial, involving enhanced alpha-adrenergic tone, thromboxane release from platelet activation, endothelin-1 signaling, and calcium channel-mediated depolarization, often compounded by mechanical endothelial injury during harvesting [2,21]. Pharmacological vasodilators are routinely applied intraoperatively and postoperatively to manage graft spasm; however, some widely used agents have notable limitations. Papaverine has been shown to injure the IMA endothelium during preparation of the graft and impair endothelium-dependent relaxation [22,23,24]. Calcium channel blockers and nitroglycerin act primarily through endothelium-independent mechanisms—blocking L-type calcium channels or directly donating NO to smooth muscle, respectively—but do not provide a combined effect on both endothelial function and smooth muscle relaxation [21,25]. An ideal antispasmodic agent would therefore combine effective smooth muscle relaxation with preservation or enhancement of endothelial NO signaling—an approach that remains largely unmet by currently available therapies.

Body Protection Compound-157 (BPC 157) is a stable pentadecapeptide derived from human gastric juice with a highly favorable safety profile in phase II clinical trials for inflammatory bowel disease and no identified lethal dose [26,27,28]. In addition to its well-established wound-healing properties across skin [29,30], gastrointestinal tract [31,32,33], tendons [34,35], muscles [36,37], and blood vessels [38], BPC 157 has attracted particular attention for its interaction with the NO system, where stimulation of NO synthesis has been identified as a central mechanism underlying its therapeutic effects across various models and species [39,40,41,42,43,44,45,46,47]. This NO-related activity extends to the vasculature: in vivo, BPC 157 reverses hyperkalemia-induced blood pressure disturbances [45], reduces portal hypertension [48,49], and counteracts both Nω-nitro-L-arginine methyl ester (L-NAME)-induced hypertension and L-arginine-induced hypotension [50,51,52,53,54]. At the molecular level, BPC 157 enhances VEGFR2 expression and rapidly phosphorylates eNOS in endothelial cells [40] and has been shown to modulate vasomotor tone of isolated rat aorta through Src–Caveolin-1 (Cav-1)–eNOS signaling, producing concentration-dependent, endothelium-dependent vasorelaxation that was abolished by L-NAME and hemoglobin [41]. However, all existing evidence on the direct vasorelaxant effects of BPC 157 derives from animal models, and whether these endothelium-dependent, NO-mediated effects translate to human conduit arteries has not been established.

This study was therefore designed to preliminarily investigate the vasorelaxant effect of BPC 157 on phenylephrine-stimulated human IMA rings obtained from patients undergoing elective CABG surgery and to characterize its concentration-dependent effects on stimulated arterial contraction. Using functional endothelial denudation and pharmacological NOS inhibition with L-NAME, we aimed to determine the contribution of the endothelial NO pathway to BPC 157-induced vasorelaxation and to provide the first translational evidence of BPC 157’s vascular activity under phenylephrine-induced (α-adrenergic) vasoconstrictive conditions in human arterial tissue, extending prior findings from animal models into a clinically relevant conduit vessel.

## 2. Materials and Methods

### 2.1. Surgical and Experimental Procedures

Patients who underwent scheduled elective triple CABG surgery were informed about the protocol of IMA harvesting, and those who agreed to participate and gave written informed consent were included in the present study. All 14 patients (all male) had been diagnosed with coronary artery disease and had severe stenosis (70–99%) in the left anterior descending, left circumflex and right coronary arteries. However, the harvested IMA remnants from two patients were not suitable for in vitro functional studies; therefore, the experimental procedures and final analyses were conducted using IMA tissue obtained from 12 patients. All patients were diagnosed recently and were not on long-term medication. Preoperative medication during hospitalization for the patients included metoprolol as a beta-blocker, perindopril as an angiotensin-converting enzyme (ACE) inhibitor and sacubitril/valsartan as an angiotensin receptor–neprilysin inhibitor (ARNI). The use of calcium channel blockers was avoided. All medication was discontinued 24 h prior to surgery. The IMA segments remaining after grafting (considered surgical waste) were used in this study. The protocol was approved by the Research Ethics Committee at Gulhane Training and Research Hospital, University of Health Sciences (Approval no: 2024-235, date: 24 April 2024). The study was conducted in accordance with the ethical standards of the Declaration of Helsinki.

Nitroglycerine exposure was avoided from 24 h prior to surgery until completion of IMA harvesting. Immediately upon collection, the IMA segments were placed in oxygenated cold Krebs–Henseleit solution of the following composition: 118.4 mM NaCl, 4.7 mM KCl, 1.2 mM KH_2_PO_4_, 1.2 mM MgSO_4_, 25.0 mM NaHCO_3_, 2.5 mM CaCl_2_ and 12.2 mM glucose, pH 7.4, and transferred to the Hacettepe University Faculty of Medicine Physiology Laboratory for in vitro functional studies. Surrounding connective tissue was dissected gently from arterial segments to prepare two arterial rings (3–4 mm) from each patient (24 rings in total). The IMA rings were randomly allocated into endothelium-intact and endothelium-denuded groups. The rings were submerged in 10 mL double-jacketed organ baths containing Krebs–Henseleit solution, maintained at 37 °C, 7.4 pH, and were gassed continuously with a mixture of 5% CO_2_ and 95% O_2_. The rings were attached to an isometric force displacement transducer (MAYFDT2, BIOPAC Systems Inc., Goleta, CA, USA) and changes in force were recorded in real time (BIOPAC MP36, BIOPAC Systems Inc., Goleta, CA, USA) under 5 mN resting tension for at least 60 min, during which the bath solution was renewed every 15 min until vascular rings reached equilibrium.

The arterial rings were employed in three consecutive protocols. First, the equilibrated rings were stimulated with 3 × 10^−6^ M phenylephrine (PheE) for 10 min, both to assess the rings’ responsiveness and obtain the maximum contractile response. The PheE stimulation was followed by 3 × 10^−6^ M acetylcholine (ACh) exposure to confirm endothelial integrity or successful endothelial removal. Two rings (one from the endothelium-intact group and one from the endothelium-denuded group, obtained from two different patients) failed to contract in response to PheE stimulation and were excluded from subsequent protocols; therefore, the final number of IMA rings analyzed was 11 in both endothelium-intact and endothelium-denuded groups.

The rings were re-equilibrated with washouts every 15 min for at least 60 min after each protocol. In the second set of experiments, to investigate the effect of BPC 157 on arterial tone, cumulative dose–response curves of BPC 157 (0.01, 0.1, 0.5, 1 mg/mL) were obtained in PheE (3 × 10^−6^ M)-stimulated rings. Each consecutive dose was applied at 10 min intervals. In the last protocol, we investigated the role of the endothelial layer in the arterial responses. After the rings were pre-incubated with the nitric oxide synthase (NOS) inhibitor L-NAME (10^−6^ M) for 10 min, the protocol was repeated as described above. At the end of the experiments, 120 mM KCl–Krebs solution was added to all baths to verify tissue viability. The drugs were added to the organ bath in a volume of 100 µL. After each procedure, baths were washed three times and waited for the rings to stabilize for 60 min.

### 2.2. Solutions and Drug

Krebs–Henseleit solution was prepared freshly in the morning of the experimental days and the chemicals used in its preparation were purchased from Merck (Merck KGaA, Darmstadt, Germany). BPC 157 (sequence: GEPPPGKPADDAGLV; molecular weight: 1419.54 g/mol) was purchased from Sigma-Aldrich Chemie GmbH (Taufkirchen, Germany). The compound was synthesized to a purity exceeding 95%, confirmed by high-performance liquid chromatography (HPLC), and was highly soluble in distilled water under physiological pH conditions. PheE, ACh and L-NAME were also purchased from Sigma-Aldrich (Taufkirchen, Germany). Stock solutions and serial dilutions of the drugs were prepared using distilled water. The concentrations of BPC 157 were determined and adjusted based on the study by Ming-Jer Hsieh et al. [41].

### 2.3. Calculations and Statistical Analysis

The recorded data were analyzed using Biopac MP36 (BIOPAC Systems, Inc., Goleta, CA, USA) data acquisition and analysis system via BSLPRO software (version 3.6.7) to measure the maximum contraction of the vascular tissue (g) and the area under the curve (AUC) over 10 min (integral/100 mg tissue weight/min). The data were first normalized to tissue weight (g/mg tissue weight), and both the maximum and AUC values were expressed as percentages (%) relative to the initial PheE stimulation. Statistical analysis was performed using IBM SPSS Statistics software version 23.0. All data were first evaluated for normal distribution using the Shapiro–Wilk test. Normally distributed data were compared between endothelium-intact and denuded groups using Student’s *t*-test. Within-group comparisons were performed using repeated-measures analysis of variance (ANOVA) followed by Tukey’s post hoc test for multiple comparisons. The maximal response (Emax) and half-maximal effective concentration (EC50) values were calculated by nonlinear regression of individual concentration-response curves using GraphPad Prism software (version 10.6.1, GraphPad Software, LLC, San Diego, CA, USA). EC50 was expressed as pEC50, defined as the negative logarithm of EC50 (pEC50 = −log(EC50)), in accordance with the literature data [55,56,57]. Data are presented as mean ± standard deviation (SD). A *p*-value < 0.05 was considered statistically significant.

An a priori power analysis was performed using G*Power 3.1.9.4 (Heinrich-Heine-Universität Düsseldorf, Düsseldorf, Germany), assuming a medium effect size (f = 0.25), α = 0.05 and power (1 − β) = 0.80. The analysis yielded a minimum total sample size of 22 with an actual power of 0.836.

## 3. Results

The demographic and clinical features of the patients included in the study (*n* = 12) are presented in Table 1.

### 3.1. Baseline Contractile Properties and Validation of Endothelial Integrity

Basal tone and PheE (3 × 10^−6^ M)-induced contraction values are summarized in Table 2 as maximum force of contraction (g/100 mg tissue weight) and AUC (integral/100 mg tissue weight/min). The basal tone of endothelium-denuded and endothelium-intact rings was comparable.

ACh (3 × 10^−6^ M) induced a pronounced endothelium-dependent relaxation in intact rings, whereas no significant relaxation was observed in endothelium-denuded preparations. The responses differed significantly between groups for both maximum force (*p* = 0.000699) and AUC (*p* = 0.000007), with comparable levels of significance when expressed as percentages of PheE-induced contraction.

### 3.2. Concentration-Dependent Vasorelaxant Effects of BPC 157 in Endothelium-Intact and Denuded IMA Rings

**Figure 1:**
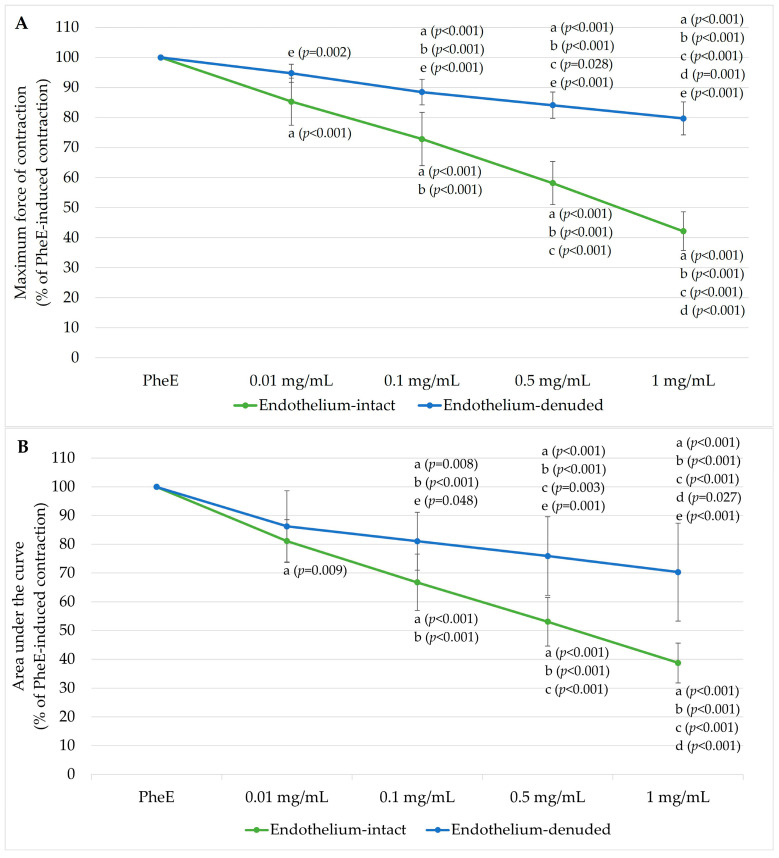
Concentration-dependent vasorelaxant effects of Body Protection Compound-157 (BPC 157) in phenylephrine (PheE)-precontracted human internal mammary artery (IMA) rings. (**A**) Maximum force of contraction and (**B**) area under the curve (AUC) in endothelium-intact (*n* = 11) and endothelium-denuded (*n* = 11) IMA rings following cumulative administration of BPC 157 (0.01–1 mg/mL). Responses are expressed as a percentage of PheE (3 × 10^−6^ M)-induced contraction. Data are presented as mean ± standard deviation (SD). Statistical annotations: a, *p* < 0.05 vs. PheE; b, *p* < 0.05 vs. 0.01 mg/mL; c, *p* < 0.05 vs. 0.1 mg/mL; d, *p* < 0.05 vs. 0.5 mg/mL; e, *p* < 0.05 vs. corresponding concentration in endothelium-intact rings.

Following PheE precontraction, cumulative BPC 157 application produced a dose-dependent reduction in vascular tone, assessed by both the maximum force of contraction and AUC (total contractile “work” over the 10-min exposure interval). This relaxant effect was more prominent in endothelium-intact rings (Figure 1). When expressed as % of PheE-induced contraction, the maximum contraction progressively decreased to 85.27 ± 7.86%, 72.83 ± 8.87%, 58.16 ± 7.19%, and 42.15 ± 6.43% at 0.01, 0.1, 0.5, and 1 mg/mL BPC 157, respectively. Similarly, AUC decreased to 81.12 ± 7.47%, 66.75 ± 9.81%, 53.06 ± 8.48%, and 38.74 ± 6.89% across the same concentrations.

In endothelium-denuded rings, BPC 157 also induced relaxation, but the magnitude was markedly attenuated: maximum contraction remained at 94.73 ± 3.03%, 88.45 ± 4.28%, 84.09 ± 4.39%, and 79.67 ± 5.48%, and AUC remained at 86.25 ± 12.40%, 81.07 ± 10.10%, 75.92 ± 13.67%, and 70.32 ± 17.04% across increasing BPC 157 doses.

Between-group differences were statistically significant for maximum force starting from 0.01 mg/mL and for AUC from 0.1 mg/mL, with the divergence becoming more pronounced at higher concentrations.

### 3.3. Nitric Oxide Synthase Inhibition with Nω-Nitro-L-Arginine Methyl Ester

**Figure 2:**
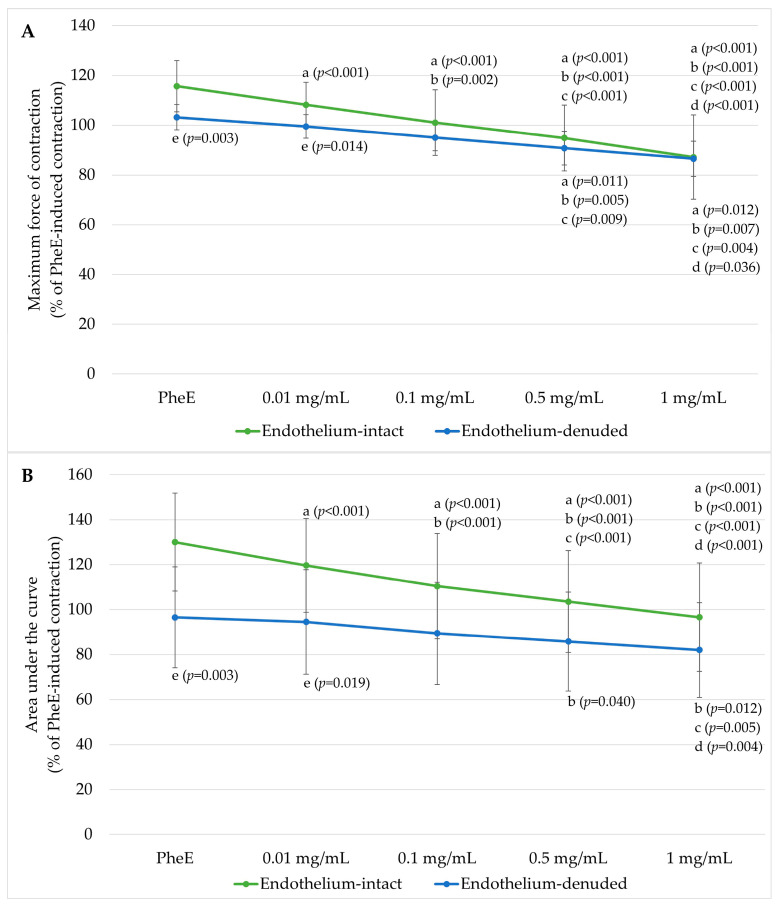
Effect of nitric oxide synthase inhibition on BPC 157-induced vasorelaxation in PheE-precontracted human IMA rings. (**A**) Maximum force of contraction and (**B**) AUC in Nω-nitro-L-arginine methyl ester (L-NAME)-pretreated endothelium-intact (*n* = 11) and endothelium-denuded (*n* = 11) IMA rings following cumulative administration of BPC 157 (0.01–1 mg/mL). Responses are expressed as a percentage of PheE (3 × 10^−6^ M)-induced contraction. Data are presented as mean ± SD. Statistical annotations: a, *p* < 0.05 vs. PheE; b, *p* < 0.05 vs. 0.01 mg/mL; c, *p* < 0.05 vs. 0.1 mg/mL; d, *p* < 0.05 vs. 0.5 mg/mL; e, *p* < 0.05 vs. corresponding concentration in endothelium-intact rings.

Pre-incubation with L-NAME markedly increased the contractile responsiveness of endothelium-intact rings (Figure 2). PheE-induced contraction increased to 115.68 ± 10.32% (maximum force of contraction) and 130.00 ± 21.79% (AUC) in intact rings, compared with 103.18 ± 5.10% and 96.54 ± 22.42% in denuded rings.

Despite NOS inhibition, BPC 157 continued to produce concentration-dependent relaxation in both groups. In L-NAME-treated intact rings, maximum contraction decreased from 108.18 ± 8.18% at 0.01 mg/mL to 87.17 ± 16.99% at 1 mg/mL, with AUC declining from 119.67 ± 20.90% to 96.57 ± 24.10%. Reductions were statistically significant from 0.01 mg/mL for both maximum contraction and AUC. In denuded rings, contraction decreased from 99.49 ± 4.73% to 86.51 ± 7.05% (maximum force of contraction) and from 94.51 ± 23.20% to 81.99 ± 21.08% (AUC), with significant reductions at 0.5 mg/mL and 1 mg/mL.

Between-group differences were significant at the PheE-only condition and at 0.01 mg/mL but progressively diminished with increasing dose. At 0.1 mg/mL, 0.5 mg/mL and 1 mg/mL, residual contraction values between intact and denuded rings became statistically comparable for both parameters. At 1 mg/mL, maximum contraction values were nearly identical (87.17% vs. 86.51%).

### 3.4. Emax and pEC50 Analysis

Emax and pEC50 values are presented in Table 3. Under control conditions, endothelium-intact rings exhibited the lowest Emax (42.15 ± 6.13), whereas endothelial removal significantly increased Emax to 79.67 ± 10.11 (*p* < 0.05). L-NAME significantly increased Emax in intact rings to 90.77 ± 19.01 (*p* < 0.05 vs. control), while the change in denuded rings was modest (86.51 ± 11.68). Under NOS inhibition, Emax values in intact and denuded rings became comparable. The pEC50 values are presented in Table 3; however, given the between-group differences in Emax and that responses were expressed as residual contraction, pEC50 values should be interpreted with caution.

## 4. Discussion

To the best of our knowledge, the present work represents the first human arterial study demonstrating a clear, concentration-dependent inhibitory effect of BPC 157 on stimulated arterial contraction together with an endothelium-dependent, NO-mediated mechanism of action. Our findings provide early mechanistic data and extend observations from existing animal models to clinically relevant human IMA tissue. The major findings are that (i) BPC 157 inhibits phenylephrine-evoked contraction in a concentration-dependent manner; (ii) this inhibitory effect is likely mediated, at least in part, through an endothelium-dependent NO pathway, although this remains indirect; and (iii) residual relaxation persists under both conditions, suggesting additional NO-independent mechanisms.

A central strength of our functional protocol is the validation of endothelial integrity prior to mechanistic inference. Endothelium-intact rings demonstrated robust ACh-induced relaxation (38.05 ± 8.17% of PheE-induced contraction), whereas endothelium-denuded rings showed markedly attenuated relaxation (86.61 ± 16.20% residual contraction), confirming effective functional endothelial removal (Table 2). The residual, modest relaxation observed in denuded rings following ACh stimulation (86.61 ± 16.20%) warrants consideration, as it may reflect either incomplete endothelial removal or, alternatively, a minor direct muscarinic effect on vascular smooth muscle. However, several lines of evidence support adequate denudation. The ACh-induced relaxation in denuded rings was dramatically attenuated compared with intact rings (*p* < 0.001). The magnitude of the residual response (~13%) is modest relative to the robust relaxation observed in intact rings. Additionally, BPC 157 responses converged between L-NAME-treated intact and denuded rings at higher concentrations. Nevertheless, we cannot entirely exclude the possibility that trace residual endothelium may have contributed, and this is acknowledged as a limitation.

The only prior study investigating BPC 157’s direct vasorelaxant effects in isolated vessels demonstrated concentration-dependent relaxation in PheE-precontracted rat aorta, with vasorelaxation reaching 28.3 ± 3.5% and 48.3 ± 3.2% at cumulative concentrations of 10 and 100 μg/mL in endothelium-intact rings, compared with only 15.2 ± 3.2% and 19.2 ± 3.5% in denuded preparations [41]. This relaxation was confirmed to be NO-mediated, as it was substantially reduced by L-NAME and hemoglobin [41]. At the molecular level, BPC 157 rapidly induced phosphorylation of Src (Tyr416), Cav-1 (Tyr14), and eNOS (Ser1177) in endothelial cells within 30 min, an effect abolished by Src inhibition with Src kinase inhibitor 1 (SKI-1) [41]. Co-immunoprecipitation analysis further revealed that BPC 157 reduced eNOS/Cav-1 binding to approximately 50% of control levels, releasing eNOS from its inhibitory interaction with Cav-1 [41]. Our human IMA data are qualitatively concordant with these findings but reveal a quantitatively more pronounced endothelial contribution: residual contraction at the highest BPC 157 concentration fell to approximately 42% in endothelium-intact rings, whereas denuded rings remained near 80%. This greater divergence likely reflects the contribution of endothelium to vasodilatation and documented abundance of eNOS in the IMA [58,59], which may amplify BPC 157’s capacity to stimulate endothelial NO production through the Src–Cav-1–eNOS cascade [41]. However, unlike the rat aorta study in which denuded vessels showed only marginal relaxation and no direct smooth muscle effect was detectable, our endothelium-denuded IMA rings exhibited a modest but statistically significant dose-dependent relaxation beginning at 0.1 mg/mL, with residual contraction decreasing from approximately 88% to 80%. This discrepancy suggests that human IMA smooth muscle may possess additional non-endothelial targets for BPC 157 and warrants further investigation.

In Figure 1, cumulative BPC 157 produced a clear concentration-dependent reduction in PheE-stimulated contraction in both groups, with maximum contraction in endothelium-intact rings falling progressively from 85.27 ± 7.86% at 0.01 mg/mL to 42.15 ± 6.43% at 1 mg/mL, and corresponding AUC values decreasing from 81.12 ± 7.47% to 38.74 ± 6.89%. This pattern aligns with vasodilation studies using other endothelium-active agents in human IMA: VEGF induces concentration-dependent, endothelium-dependent relaxation in human IMA, attenuated by NOS inhibition and completely abolished by endothelial removal, with the relaxation mediated through both NO and PGI_2_ pathways via KDR/VEGFR2 [16,17]. VEGFR2 is physically associated with Cav-1 in endothelial caveolae. BPC 157 has been shown to enhance VEGFR2 expression and activate downstream protein kinase B (Akt)/eNOS signaling [40]. BPC 157 may also engage the same VEGFR2-linked endothelial pathway to augment NO availability through VEGFR2–Src–Cav-1–Akt–eNOS signaling, producing stronger relaxation when the endothelium is intact. However, VEGF-induced relaxation in human IMA is entirely endothelium-dependent—abolished by endothelial denudation, and pharmacologically eliminated only by combined NOS inhibition, cyclooxygenase inhibition, and NO scavenging with oxyhemoglobin [16,17]. In our study, BPC 157 retains significant residual relaxation in denuded rings, indicating additional direct smooth muscle mechanisms beyond endothelial pathways.

The persistence of significant, dose-dependent relaxation in endothelium-denuded rings indicates that BPC 157 is not exclusively endothelium-dependent in human IMA, and several mechanisms may contribute. In endothelium-denuded rings, where endothelium-derived mediators are absent, the statistically significant progressive relaxation observed from 0.1 mg/mL onward—reaching approximately 80% residual contraction at the highest concentration—supports a direct smooth muscle mechanism. Although the precise pathway requires further molecular investigation and current literature on this effect is limited, plausible mechanisms include reduced Ca^2+^ influx through voltage-gated channels or decreased myofilament Ca^2+^ sensitivity via inhibition of the Ras homolog family member A (RhoA)/ROCK pathway, which normally sustains contraction by suppressing myosin light chain phosphatase activity independently of intracellular Ca^2+^ levels [60]. The possibility of residual endothelial activity contributing to this response cannot be entirely excluded without direct molecular confirmation.

The mechanistic centerpiece of our study is the L-NAME experiment (Figure 2). Pre-incubation with L-NAME markedly augmented contractile responsiveness of endothelium-intact rings, consistent with removal of basal NO-mediated vasodilatory restraint—a phenomenon well characterized in human IMA [58]. Under L-NAME conditions, PheE-induced contraction was numerically higher in intact than in denuded rings, a discrepancy that likely reflects the fact that mechanical denudation removes the endothelium entirely—eliminating all endothelial modulators including NO, PGI_2_, and EDHF—whereas pharmacological NOS inhibition selectively suppresses NO synthesis while other endothelial mediators remain. Most importantly, under NOS inhibition, the difference between intact and denuded rings progressively diminished, and from 0.1 mg/mL concentration, the responses converged such that residual contraction became statistically comparable. This convergence is also reflected in the Emax analysis: under control conditions, Emax was markedly lower in endothelium-intact rings (42.15 ± 6.13) compared with denuded rings (79.67 ± 10.11; *p* < 0.05), whereas L-NAME significantly increased Emax in intact rings to 90.77 ± 19.01, rendering it comparable to denuded rings (86.51 ± 11.68). Taken together, these findings support a probable contribution of endothelium-derived NO to BPC 157-induced vasorelaxation; however, this evidence remains indirect, as it is based on pharmacological NOS inhibition rather than direct molecular assessment. Therefore, NO cannot be considered the sole or final mediator, and confirmation requires direct evaluation of NO production, eNOS activation, and related signaling pathways.

Notably, endothelium-denuded rings pre-incubated with L-NAME exhibited a limited and statistically significant vasorelaxant response to higher concentrations (0.5 and 1 mg/mL) of BPC 157. This pattern indicates that, in the absence of both functional endothelium and nitric oxide synthesis, BPC 157 exerts only a weak direct effect on vascular smooth muscle, requiring higher concentrations to achieve measurable relaxation. This finding may suggest the presence of a minor NO component, potentially arising from constitutive neuronal NOS (nNOS) or inducible NOS (iNOS) activity within vascular smooth muscle [61,62], or from trace residual endothelium. Whether this contribution is physiologically relevant remains uncertain and requires further investigation.

In L-NAME-treated endothelium-intact rings, where the endothelium is preserved but NOS is inhibited, additional endothelium-derived mediators may account for the residual dose-dependent relaxation. EDHF pathways involving BKCa channels are plausible contributors, as EDHF in human IMA has been identified as 11,12-epoxyeicosatrienoic acid acting via BKCa channels [14]. PGI_2_-related mechanisms may also participate, since VEGF-induced relaxation in IMA involves both NO and PGI_2_ pathways [16], and BPC 157 shares signaling elements with the VEGF pathway. Additionally, modulation of endothelin signaling may play a role, as BPC 157 has previously been associated with endothelin pathway modulation [38,63]. Future studies employing selective inhibitors—iberiotoxin, indomethacin, and isoform-selective NOS inhibitors—will be essential for dissecting the relative contributions of these pathways.

Regarding the concentration–response analysis, Emax is the most informative parameter for between-group comparison because it directly reflects residual contraction at maximal BPC 157 effect. The pEC50 values, although reported for completeness, should be interpreted with caution rather than as standalone indices of vasorelaxant sensitivity, as they are substantially influenced by differences in maximal efficacy and curve geometry when responses are expressed as residual contraction. Future studies using a denser concentration range and direct relaxation analysis from the precontracted baseline will allow more rigorous pharmacological characterization.

From a translational perspective, the vasorelaxant profile of BPC 157 appears mechanistically distinct from currently used approaches to the management of IMA spasm during CABG. Papaverine, a commonly used intraoperative antispasmodic, effectively relieves graft spasm but has also been reported to injure the IMA endothelium, at least in part because of its acidic formulation [22,23]. In addition, higher papaverine concentrations have been associated with greater endothelial damage in ex vivo arterial graft studies, including reduced cluster of differentiation 34 (CD34)-positive endothelial coverage [64]. Calcium channel blockers such as amlodipine, by contrast, produce vasorelaxation predominantly through direct inhibition of L-type calcium influx in vascular smooth muscle and are therefore largely independent of endothelial mediation [25]. In our study, BPC 157 exerted its predominant vasorelaxant effect through an endothelium-dependent NO pathway, with only a modest residual response after endothelial denudation or NOS inhibition. These findings do not establish clinical superiority over existing agents, nor do they demonstrate direct endothelial protection. However, they do suggest that BPC 157 may warrant further investigation as an antispasmodic candidate whose effects are mediated, at least in part, through endogenous endothelial signaling rather than solely through direct smooth muscle blockade. Given the established contribution of endothelial integrity and NO bioavailability to the favorable vasodilatory, antithrombotic, and antiatherogenic properties of the IMA graft, this mechanistic profile could be clinically relevant if confirmed in vivo [2,65]. Whether BPC 157 can prevent perioperative IMA spasm while preserving endothelial integrity and graft function remains to be determined in dedicated in vivo and clinical studies.

The interpretation of these findings should also consider the characteristics of the study population and tissue source. All included patients were male, and vascular reactivity, as well as endothelial biology, are known to exhibit sex-related differences, which may limit extrapolation to female patients. In addition, the internal mammary artery samples were obtained from individuals with advanced coronary artery disease and multiple cardiovascular risk factors. While this context increases clinical relevance, it also suggests that baseline endothelial function and vascular responsiveness may already be altered. Accordingly, the observed effects of BPC 157 may reflect disease-modified vascular biology rather than responses representative of normal, healthy vessels.

The concentration range (0.01–1 mg/mL, equivalent to 10–1000 μg/mL) warrants discussion. The two lower concentrations (10 and 100 μg/mL) directly match those at which Hsieh et al. demonstrated endothelium-dependent vasorelaxation and eNOS phosphorylation in rat aorta [41], enabling cross-species comparison, while the two higher concentrations (500 and 1000 μg/mL) were included to determine maximal efficacy in human IMA. These organ bath concentrations substantially exceed systemic plasma levels; pharmacokinetic data show that intramuscular injection of 100 μg/kg BPC 157 in rats yields peak plasma concentrations in the nanogram-per-milliliter range with a half-life under 30 min [66]. However, supraphysiological concentrations are standard in ex vivo organ bath pharmacology to characterize concentration-response relationships [67]. Moreover, in the clinical CABG setting, topical or intraluminal application of vasodilators to IMA grafts is routine and would achieve substantially higher local tissue concentrations than systemic dosing. Whether BPC 157 could be effectively and safely applied topically to IMA grafts requires dedicated in vivo dose-finding studies.

Several limitations should be considered. Importantly, the clinical translation of these findings is inherently limited, as the experiments were performed in isolated human arterial rings under ex vivo conditions. This model does not replicate key in vivo physiological factors, including pulsatile blood flow, circulating blood cells, systemic inflammatory responses, neurohumoral regulation, and perioperative surgical stress. Therefore, the present results should be interpreted as mechanistic functional evidence only and cannot directly demonstrate the effects of BPC 157 in patients undergoing CABG. Another limitation is the potential for confounding by patient-related factors. Clinical characteristics such as smoking status, hypertension, diabetes mellitus, hyperlipidemia, and ventricular function are known to influence endothelial biology and vascular reactivity. Although these variables were documented, no adjustment or stratified analysis was performed. Therefore, the observed vascular responses may have been influenced, at least in part, by these uncontrolled confounders. Medication-related confounding should also be considered. Although cardiovascular medications were discontinued prior to surgery, many patients had been receiving chronic treatments, including beta-blockers, ACE inhibitors, ARNI, and statins. The biological effects of these agents on endothelial function and vascular reactivity may persist beyond short-term discontinuation. Therefore, incomplete washout of chronic medication could have influenced vascular tone and NO-related responses in the present study. The external validity of the present findings is also limited. The arterial samples were obtained from a relatively small and highly specific patient population consisting exclusively of male individuals with advanced coronary artery disease. As such, the results may not be generalizable to female patients, to individuals with healthier vascular profiles, or to other vascular beds with different structural and functional properties.

Another limitation relates to pathway specificity. Although the present study primarily implicates NO based on L-NAME inhibition, other endothelium-dependent pathways, including PGI_2_ and EDHF, were not directly investigated. In addition, potential smooth muscle mechanisms—such as modulation of calcium influx, potassium channel activity, or downstream cGMP signaling—were not specifically evaluated. Therefore, the proposed mechanism remains incomplete, and alternative or complementary pathways may contribute to the observed vasorelaxant effects. Direct molecular measurements were also absent. Although pathways involving eNOS, Cav-1, VEGFR2, Src, and Akt signaling are discussed, none of these were directly assessed in the human arterial samples. Therefore, the proposed molecular mechanisms are based on indirect functional evidence and prior literature rather than direct experimental confirmation in this study.

A single vasoconstrictor agonist was used in the study. Although use of PheE is a well-established model for α-adrenergic-mediated contraction, internal mammary artery spasm in the clinical setting is multifactorial and involves additional mediators, including thromboxane-related pathways, endothelin-1, and other vasoactive stimuli. Therefore, the present findings may not fully represent the complex biology of graft spasm.

An additional limitation concerns the endothelial removal model. Mechanical denudation does not selectively eliminate NO signaling but removes the entire endothelial layer, thereby abolishing all endothelium-derived mediators, including PGI_2_ and EDHF. Moreover, the denudation process may alter the vessel surface and vascular responsiveness in a non-physiological manner. Consequently, endothelial denudation and NOS inhibition represent fundamentally different experimental models, and comparisons between them should be interpreted with caution.

Potential tissue selection bias should be considered. Two harvested IMA samples were excluded due to insufficient size, and two additional rings were excluded because they failed to exhibit adequate contractile responses, potentially related to surgical excision-associated damage. Although these exclusions were technical in nature and no systematic differences were identified, the preferential inclusion of structurally intact and functionally responsive samples may have introduced a degree of selection bias.

The final sample size (*n* = 11 rings per group) was determined by tissue availability from elective CABG procedures; however, an a priori power analysis confirmed that this sample size meets the minimum requirement of 22 total subjects (11 per group) for adequate statistical power. Moreover, consistent within-group dose–response effects and significant between-group differences across multiple concentrations further support the reliability of the observed findings, and the sample size is comparable to other studies in the literature [68,69,70]. Statistical transparency is also limited by the small sample size and multiple comparisons. Although repeated-measures ANOVA with post hoc testing was applied, assumption testing may be underpowered in small samples. In addition, multiple pairwise comparisons increase the risk of type I error, and no more conservative adjustment beyond standard post hoc procedures was applied. Accordingly, the findings should be interpreted cautiously. An additional methodological limitation also relates to the unit of analysis. As multiple arterial rings were obtained from the same patients, the assumption of statistical independence between observations may not be fully valid, introducing a risk of pseudoreplication. This may overestimate the effective sample size and influence variance estimates. As no statistical adjustment for within-patient clustering was performed, the results should be interpreted with caution, and future studies using patient-level or mixed-effects analyses are warranted.

## 5. Conclusions

The present study demonstrates that BPC 157 produces a concentration-dependent inhibition of PheE-induced contraction in human IMA rings. This vasorelaxant effect is more pronounced in endothelium-intact preparations and is attenuated following NOS inhibition, suggesting a probable contribution of an endothelium-dependent NO pathway based on indirect functional evidence, although this mechanism requires molecular confirmation. Endothelial integrity enhances the maximal efficacy of BPC 157-induced relaxation. The persistence of relaxation in endothelium-denuded and L-NAME-treated rings indicates additional NO-independent mechanisms, particularly at higher concentrations. These findings were obtained under controlled ex vivo conditions using PheE-induced vasoconstriction and should be interpreted within this context. Although the study provides preliminary functional evidence that BPC 157 may modulate vascular tone in human IMA, it does not demonstrate clinical benefit in terms of graft patency, perioperative vasospasm prevention, myocardial perfusion, or patient outcomes. Overall, the results are exploratory and hypothesis-generating rather than mechanistically definitive, and further studies incorporating direct NO measurements, molecular signaling analyses, expanded pharmacological evaluation, larger and more diverse samples, and in vivo validation are required to clarify mechanisms and determine clinical relevance.

## Figures and Tables

**Table 1:** Baseline demographic and clinical characteristics of the study population. Data are presented as mean ± standard deviation (SD) for continuous variables and as number (percentage) for categorical variables. Body mass index (BMI), left ventricular ejection fraction (LVEF), angiotensin-converting enzyme (ACE) inhibitor and angiotensin receptor–neprilysin inhibitor (ARNI).

Variable	Value (*n* = 12)
Age (years)	60.3 ± 7.5
BMI (kg/m^2^)	27.2 ± 3.5
Hypertension	5 (41.7%)
Diabetes Mellitus	3 (25%)
Hyperlipidemia	5 (41.7%)
Current smokers	9 (75%)
LVEF (%)	53.8 ± 5.7
Beta-blocker use	12 (100%)
ACE inhibitor use	3 (25%)
ARNI use	2 (16.7%)
Statin use	5 (41.7%)

**Table 2:** Basal contractile tone, phenylephrine (PheE)-induced contraction, and acetylcholine (ACh)-induced relaxation in human internal mammary artery (IMA) rings. Basal tone, PheE (3 × 10^−6^ M)-stimulated contraction, and ACh (3 × 10^−6^ M)-induced relaxation are presented as maximum force of contraction (g/100 mg tissue weight) and as area under the curve (AUC; integral/100 mg tissue weight/min) measured over a 10-min period. Results are expressed both as absolute values and as percentages of the corresponding PheE-induced maximum response. Data are shown as mean ± SD. Comparisons were made between endothelium-intact and endothelium-denuded rings. * *p* < 0.001 vs. endothelium-intact group.

Parameter	Experimental Condition	Outcome Measure (Unit)	Endothelium-Intact Group (*n* = 11)	Endothelium-Denuded Group (*n* = 11)
Maximum force of contraction	Basal Tone	g/100 mg tissue weight	3.39 ± 0.90	3.51 ± 0.58
		% of PheE-induced contraction (%)	33.38 ± 9.78	29.87 ± 7.60
	Response to PheE stimulation	g/100 mg tissue weight	10.86 ± 3.67	12.43 ± 3.77
		% of PheE-induced contraction (%)	100.00	100.00
	Response to ACh stimulation	g/100 mg tissue weight	4.20 ± 1.65	11.08 ± 4.03 *
		% of PheE-induced contraction (%)	38.05 ± 8.17	86.61 ± 16.20 *
Area Under the Curve	Basal Tone	integral/100 mg tissue weight/min	44.02 ± 15.95	46.28 ± 15.64
		% of PheE-induced contraction (%)	46.58 ± 19.79	42.27 ± 14.21
	Response to PheE stimulation	integral/100 mg tissue weight/min	101.94 ± 28.85	114.07 ± 34.98
		% of PheE-induced contraction (%)	100.00	100.00
	Response to ACh stimulation	integral/100 mg tissue weight/min	34.32 ± 12.69	97.02 ± 38.86 *
		% of PheE-induced contraction (%)	33.29 ± 6.78	82.03 ± 16.31 *

**Table 3:** Concentration–response parameters of Body Protection Compound-157 (BPC 157) in human IMA rings. Emax represents the maximal contraction expressed as a percentage of PheE-induced contraction following cumulative BPC 157 application. pEC50 denotes the negative logarithm of the concentration required to achieve 50% of the maximal effect, calculated by nonlinear regression analysis of individual concentration–response curves. Data are presented as mean ± SD. Endothelium-intact and endothelium-denuded rings were analyzed under control conditions and after pre-incubation with the nitric oxide synthase inhibitor Nω-nitro-L-arginine methyl ester (L-NAME). * *p* < 0.05 vs. corresponding control group; ** *p* < 0.05 vs. endothelium-intact control group.

	Endothelium-Intact Group (*n* = 11)		Endothelium-Denuded Group (*n* = 11)	
	Control	L-NAME Pre-Incubated	Control	L-NAME Pre-Incubated
Emax	42.15 ± 6.13	90.77 ± 19.01 *	79.67 ± 10.11 **	86.51 ± 11.68
pEC50	0.523 ± 0.268	2.265 ± 1.143 *	2.657 ± 0.549 **	2.259 ± 0.510

## Data Availability

Additional data is unavailable due to ethical restrictions and privacy.
